# Effects of pentoxifylline on whole blood IL‐2 and IFN‐gamma gene expression in normal dogs

**DOI:** 10.1002/vms3.204

**Published:** 2019-10-16

**Authors:** Evangel Kummari, Andres Gibbs, Caitlin Riggs, Claire Fellman, John Stokes, John Thomason, Robert Wills, Andrew Mackin, Todd Archer

**Affiliations:** ^1^ Department of Basic Sciences Mississippi State University College of Veterinary Medicine Mississippi State MS USA; ^2^ Department of Clinical Sciences Mississippi State University College of Veterinary Medicine Mississippi State MS USA; ^3^ Department of Pathobiology and Population Medicine Mississippi State University College of Veterinary Medicine Mississippi State MS USA; ^4^ Present address: Department of Clinical Sciences Tufts University 200 Westboro Road North Grafton MA 01536 USA; ^5^ Present address: Randall Veterinary Hospital Byram MS USA

**Keywords:** cyclosporine, cytokine, dog, interferon‐γ, interleukin‐2, lymphocytes, pentoxifylline

## Abstract

**Background:**

Pentoxifylline (PTX) is a methylxanthine phosphodiesterase inhibitor that is used as a hemorrheologic and anti‐inflammatory agent in veterinary and human medicine. In human studies, PTX has been shown to decrease T‐cell production of cytokines such as IL‐2 and IFN‐γ. A RT‐qPCR assay to measure activated T‐cell gene expression of IL‐2 and IFN‐γ has been validated in dogs.

**Objectives:**

The goal of this study was to utilize this assay to investigate the effects of PTX on in vitro cytokine gene expression in canine whole blood.

**Methods:**

Whole blood from seven healthy dogs was collected and incubated with various concentrations of PTX for 1 hr before activation. PTX concentrations spanned and exceeded blood concentrations achieved when administered at clinically relevant dosages (1, 2, 10, 50 and 200 μg/ml). Cyclosporine was used at a concentration of 500 ng/ml as a positive control. All blood samples, including untreated activated baseline samples, were then activated with phorbol myristate acetate and ionomycin for 5 hrs.

**Results:**

Analysis of activated whole blood by RT‐qPCR revealed that there was not a significant suppression of IL‐2 or IFN‐γ gene expression at any concentration of PTX when evaluating ΔCt values. All samples exposed to cyclosporine showed significant changes from untreated activated baseline samples, demonstrating marked suppression as the positive control. Cytokine expression, presented as a percentage of untreated activated baseline samples, was also evaluated. After exposure to the highest concentration of PTX (200 μg/ml), median percentage cytokine expression was suppressed to just below 50% of baseline values. This concentration, however, is much higher than blood concentrations reported to be achieved at standardly used pentoxifylline doses.

**Conclusions:**

PTX does not appear to significantly suppress T‐cell cytokine production in samples from most dogs at clinically relevant drug concentrations. Further testing is needed to establish the full effects of PTX on the immune system in dogs.

## Graphical abstract

Pentoxifylline is a methylxanthine phosphodiesterase inhibitor that is used as a hemorrheological and anti‐inflammatory agent in veterinary and human medicine. In human studies, it has been shown to decrease T‐cell production of IL‐2 and IFN‐gamma, but the effects in dogs is unknown. In this in‐vitro study in dogs, pentoxifylline did not appear to significantly suppress T‐cell cytokine production in samples from most dogs at clinically relevant drug concentrations.

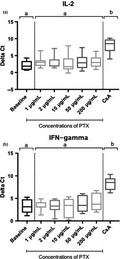

## 1 INTRODUCTION

Pentoxifylline (PTX), a methylxanthine derivative, is an immunomodulatory agent that non‐selectively inhibits phosphodiesterase enzymes (PDE), and that is used extensively in humans for vascular and inflammatory diseases, with multiple effects on white blood cells, red blood cells and platelets (Samlaska & Winfield, 1994). In T lymphocytes, macrophages and eosinophils, the main affected isoenzyme is PDE‐4, an enzyme involved in cell activation and secretion (Marsella & Olivry, 2001). Proposed effects of PTX on white blood cells include decreased neutrophil function, increased leukocyte chemotaxis, decreased monocyte tumour necrosis factor‐α (TNF‐α) production, decreased leukocyte response to TNF‐α, decreased leukocyte response to IL‐1, inhibition of B‐ and T‐cell activation and decreased natural killer cell activity (Marsella & Olivry, 2001; Samlaska & Winfield, 1994). Pentoxifylline has well‐established immune‐modulatory effects both in vitro and in vivo, via multiple proposed mechanisms of action, including a dose‐dependent increase in intracellular cyclic adenosine monophosphate (cAMP) within mononuclear and polymorphonuclear cells (Samlaska & Winfield, 1994; Semmler et al., 1993).

In vitro human studies have shown that expression of interleukin 2 (IL‐2), interferon‐gamma (IFN‐γ) and TNF‐α is inhibited when human peripheral blood mononuclear cells (PBMCs) are exposed to PTX (Benbernou, Esnault, Potron, & Guenounou, 1995; Bienvenu et al., 1995; Rieneck, Diamant, Haahr, Schonharting, & Bendtzen, 1993; Thanhauser et al., 1993; Wang, Tam, Hughes, Rath, & Sen, 1997). Pentoxifylline in combination with dexamethasone and cyclosporine exerts synergistic immunomodulatory effects on healthy donor human lymphocytes (Briggs et al., 1998), and inhibits the release of IL‐2, IFN‐γ and, to a lesser degree, TNF‐α from human PBMCs (Funk, Ernst, Schonharting, & Zabel, 1995). In one in vitro study, production of IL‐2 by human mononuclear cells was reduced following exposure to a PTX concentration of 10 µg/ml, and completely blocked at a PTX concentration of 50 µg/ml. In this same study, IFN‐γ was also inhibited in a concentration‐dependent manner by PTX, and PTX concentrations exceeding 100 µg/ml completely blocked IFN‐γ production (Böhle et al., 2000). Various human studies have also demonstrated the effects of PTX‐mediated immunomodulation in disease states, including decreased TNF‐α in AIDS patients (Fazely, Dezube, Allen‐Ryan, Pardee, & Ruprecht, 1991), inhibition of acute HIV‐1 replication in human T cells by reducing TNF‐α, IFN‐γ, and interleukin‐10 (IL‐10) production (Navarro et al., 1996), and improved cachexia in cancer patients (Dezube, Sherman, Fridovich‐Keil, Allen‐Ryan, & Pardee, 1993). PTX significantly inhibits production of the pro‐inflammatory cytokines IFN‐γ and interleukin‐17 (IL‐17) while increasing anti‐inflammatory IL‐10 production, as well as reducing lymphocyte proliferation, in type 1 diabetic mice (Malekifard, Delirezh, Hobbenaghi, & Malekinejad, 2015).

In clinical veterinary medicine, PTX is used in dogs for the treatment of canine familial dermatomyositis, contact allergy, canine atopic dermatitis, lick granulomas and symmetric lupoid onychodystrophy, and for vasculitis and other vasculopathies (Marsella, 2014; Marsella & Nicklin, 2000; Mueller, Rosychuk, & Jonas, 2003; Singh, Dimri, Saxena, & Jadhav, 2010). Pentoxifylline has also been used to treat diseases that feature tissue necrosis, such as spider bites (Marsella, 2014; Singh et al., 2010). Two PTX pharmacokinetic studies have been published in dogs using the currently available extended‐release oral PTX formulation and an intravenous form prepared by the authors for their studies. One study using eight normal dogs demonstrated a Cmax of 10.67 ± 2.69 μg/ml after intravenous administration of PTX (8 mg/kg as a single injection) and a Cmax of 1.73 ± 0.87 μg/ml after oral administration of PTX (30 mg/kg as a single oral dose; Rees et al., 2003). In a second pharmacokinetic study using seven healthy dogs, the highest PTX blood concentration seen in the dogs when PTX was administered intravenously (15 mg/kg as a single injection) was 14.3 μg/ml. The highest PTX blood concentration seen in dogs when PTX was administered orally (15 mg/kg orally every 8 hr for 5 days) was 1.47 μg/ml (Marsella, Nicklin, Munson, & Robert, 2000).

Therapeutic drug monitoring of cyclosporine in dogs can include pharmacodynamic assessment of activated whole blood IL‐2 and IFN‐γ expression. When PTX is used concurrently with cyclosporine, it may have the potential to affect interpretation of pharmacodynamic assays and impact cyclosporine dosing recommendations. The goal of our current study was to determine if exposure to PTX in vitro influenced IL‐2 and IFN‐γ expression in canine whole blood. Our hypothesis was that exposure to PTX would lead to decreased expression of IL‐2 and IFN‐γ in activated canine whole blood, as reported in human studies, when tested in vitro at concentrations achieved at clinically relevant dosages.

## 2 MATERIALS AND METHODS

### 2.1 Blood sample collection

Seven Walker hounds, members of a healthy research colony, were used for this study. Whole blood samples were collected from the jugular vein into heparinized tubes using a Vacutainer collection system. Prior to the study, the health status of each dog was evaluated through a physical examination, complete blood count, serum biochemistry profile, urinalysis, faecal flotation and heartworm testing, with no clinically apparent abnormalities noted in any dog. The dogs were not exposed to any medications or vaccinations for at least one month prior to the start of the study. All procedures were performed in accordance with the National Institutes of Health Guide for the Care and Use of Laboratory Animals, and study protocols were approved by the Institutional Animal Care and Use Committee. All animals used in this project were housed in the AAALAC accredited facilities.

### 2.2 Blood incubation and T‐cell activation

Blood samples were incubated with different concentrations of PTX (Sigma): 1, 2, 10, 50 and 200 μg/ml. Blood samples were also incubated with cyclosporine A (Sigma) at 500 ng/ml (a concentration known to suppress IL‐2 and IFN‐γ cytokine production) as a positive control (Fellman et al., 2011). A standard RNA sample was used across all plates, from a normal dog, and in which the Ct value of the standard sample was the approximately the same on all plates. Blood samples exposed to drug were incubated for 1 hr at 37°C and 5% CO_2_. After this, all blood samples were activated prior to RNA extraction with 12.5 ng/ml of phorbol myristate acetate (PMA; Sigma) and 0.8 μM of ionomycin (Sigma) as previously described (Riggs et al., 2013). All samples were then incubated for 5 hr at 37°C and 5% CO_2_.

### 2.3 RNA extraction and cytokine gene expression quantification

RNA was extracted using a previously published protocol (Riggs et al., 2013). Total RNA was isolated from 1 ml of heparinized whole blood using a QIAamp RNA Blood Mini Kit (Qiagen, Cat. No. 52,304). Genomic DNA was removed from the samples using an on‐column DNase (27.27 Kunitz units) treatment (Qiagen, Cat. No. 79,254). Samples were then stored at −80°C until further analysed. The concentration and purity of the RNA were measured by a Nanodrop ND‐1000 spectrophotometer using ND‐1000 V3.3.0 software (NanoDrop Technologies).

The results of cytokine quantitative reverse transcription polymerase chain reaction (RT‐qPCR) data were evaluated by comparing samples exposed to PTX and cyclosporine to untreated activated baseline samples using previously published protocols (Riggs et al., 2013). Briefly, a SuperScript™ III Platinum^®^ SYBR^®^ Green One‐Step kit with ROX as a reference dye (Invitrogen, Cat no. 11736‐059) was used to quantify expression of the genes of interest (IL‐2 and IFN‐γ) as well as expression of the housekeeping gene GAPDH, with the primers used being based on GenBank sequences as previously published by Kobayashi, Momoi, and Iwasaki (2007). All reactions were run on a Stratagene Mx3500P Multiplex Quantitative PCR system (Agilent Technologies) using MxPro software. The RT‐qPCR reaction was performed with a final volume of 20 µl containing a total of 30 ng of template RNA and 200 nM of each primer. The following thermal profile was used: 50°C for 3 min, 95°C for 5 min, then 40 cycles of 95°C for 15 s and 60°C for 30 s followed by a melting analysis that comprised 95°C for 15 s, 60°C for 1 min, after which the ramp speed was decreased from 1.667 to 0.01667°C/s and data are collected continuously until it reached 95°C. This temperature was then held for 30 s, and finally held at 60°C for 15 s. All samples were run in triplicate, while non‐template controls were run in duplicate. Delta Ct (ΔCt) values of treatment samples were compared to ΔCt value of untreated activated baseline samples where ΔCt = Ct_GOI_ − Ct_norm_, in which GOI is the gene of interest and norm is the reference gene. The 2^−∆∆Ct^ method was used to determine the relative change in expression using GAPDH as a reference gene where ΔΔCt = (Ct_GOI_ − Ct_norm_)_treated_ − (Ct_GOI_ − Ct_norm_)_pre‐treatment_ (Livak & Schmittgen, 2001). Cytokine gene expression in treated samples are presented as a percentage compared to untreated activated baseline samples, where untreated activated baseline samples represent 100% gene expression for IL‐2 and IFN‐γ. Relative gene expression was calculated using the formula: (2^−ΔΔCt^)* 100%.

### 2.4 Statistical methods

The effect of treatment with PTX on cytokine levels was assessed using mixed model linear regression with PROC MIXED in SAS for Windows 9.4 (SAS Institute, Inc). Separate models were fit for IL‐2 ΔCt and IFN‐γ ΔCt. In each model, treatment was included as a fixed effect with the cytokine level in untreated activated samples included as a covariate. Dog identity was included as a random effect. If significant treatment effects were determined, differences in least squares means between each of the PTX treatments and either untreated activated or cyclosporine treatments as well as between untreated activated and cyclosporine treatments were compared using an LSMESTIMATE statement with the simulate adjustment of *p*‐values for multiple comparisons. The distribution of the conditional residuals was evaluated for each outcome to ensure the assumptions of normality and homoscedasticity for the statistical method had been met. An alpha level of 0.05 was used to determine statistical significance for all methods.

## 3 RESULTS

### 3.1 RT‐qPCR

**Figure 1:**
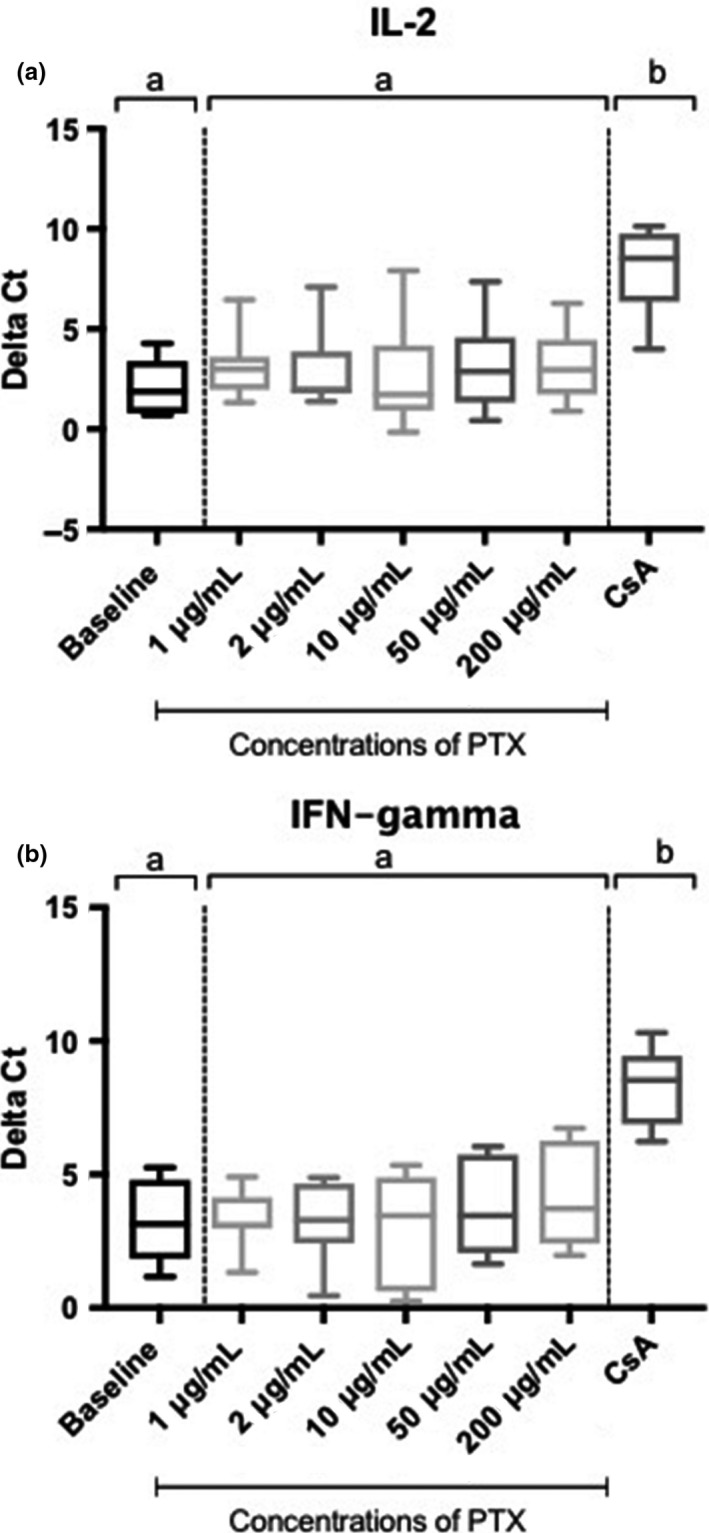
Box plots of activated whole blood IL‐2 (a) and IFN‐γ (b) mRNA expression for seven healthy Walker hounds. The effects of pentoxifylline at five different concentrations are presented, as well as the effects of cyclosporine as the positive control. Expression is presented as ∆Ct values where ΔCt = Ct_GOI_–Ct_norm_, in which GOI is the gene of interest and norm is the reference gene. The line within each box denotes the median, box edges represent the first and third quartiles, and whiskers extend to maximum and minimum values. Samples that share the same letter are not significantly different (*p* > .078). Cyclosporine was significantly different from untreated activated baseline samples (*p* < .001) and all PTX concentrations (*p* < .001). (IL‐2 = interleukin‐2, IFN‐γ = interferon‐gamma, PTX = pentoxifylline, CsA = cyclosporine)

**Figure 2:**
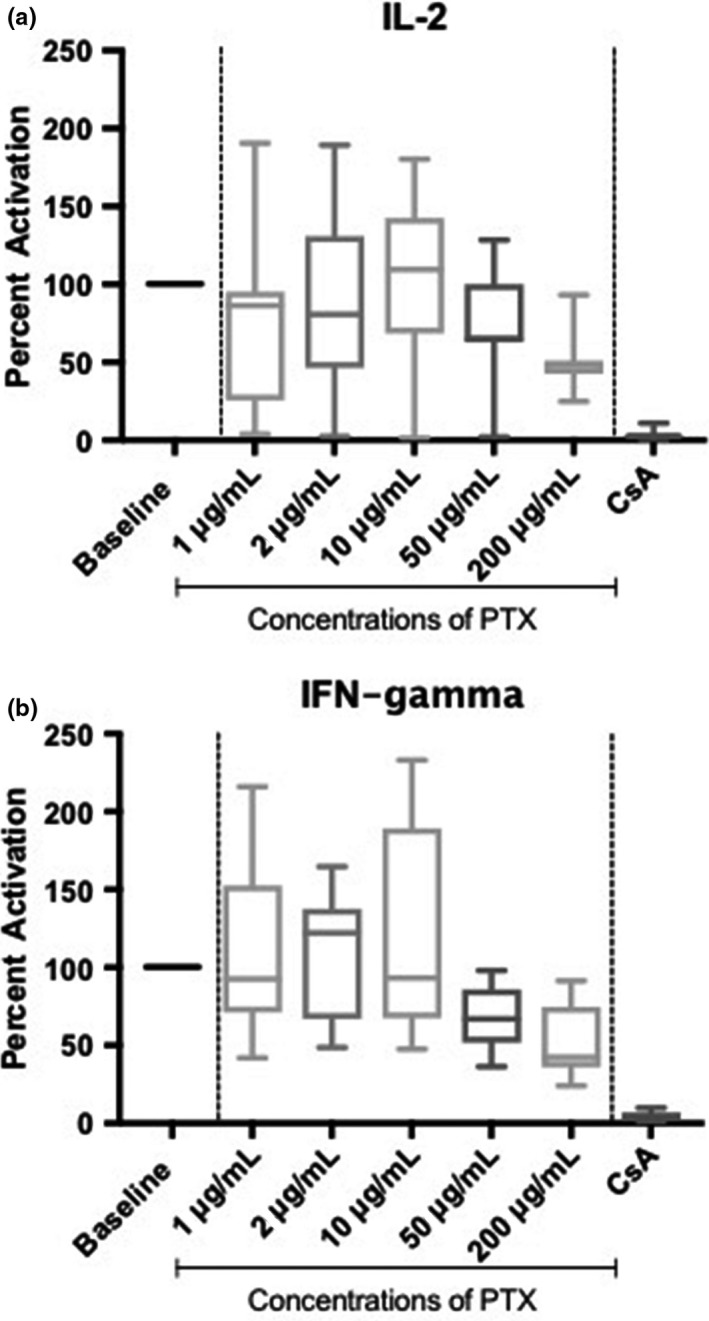
Box plots of activated whole blood IL‐2 (a) and IFN‐γ (b) mRNA expression presented as a percentage of untreated activated baseline samples, in which the untreated activated baseline samples represent 100% cytokine production, from seven healthy Walker hounds. The effect of pentoxifylline at five different concentrations are presented, as well as the effects of cyclosporine as the positive control. The line within each box denotes the median, box edges represent the first and third quartiles, and whiskers extend to maximum and minimum values. Bottom whiskers at all PTX concentrations reflect 50% or more suppression of cytokine expression in one particular dog. (IL‐2 = interleukin‐2, IFN‐γ = interferon‐gamma, PTX = pentoxifylline, CsA = cyclosporine)

The RT‐qPCR results are presented in Figures 1 and 2 for IL‐2 (a) and IFN‐γ (b). In Figure 1, ∆Ct values are presented. When comparing the ∆Ct values of the untreated activated baseline samples to the samples treated with PTX, a significant difference was not seen for either cytokine at any of the PTX concentrations (*p* > .078), demonstrating that there was not a significant change in values from baseline to treatment with PTX. When comparing the ∆Ct values of the samples exposed to PTX to the samples treated with cyclosporine, there was a significant difference for both cytokines at all of the PTX concentrations (*p* < .001), demonstrating a significant suppression of cytokines when blood was treated with cyclosporine compared to PTX. When comparing ∆Ct values for the untreated activated baseline samples to the samples treated with cyclosporine, a significant difference was seen for both cytokines demonstrating a significant suppression of cytokines when blood was treated with cyclosporine (*p* < .001). Statistical analysis was completed using the ∆Ct values.

Cytokine expression, presented as a percentage of untreated activated baseline samples, was also evaluated, and is shown in Figure 2. An untreated activated baseline sample represents 100% cytokine production. After exposure to the highest concentration of PTX (200 μg/ml), median percentage cytokine expression was suppressed to just below 50% of baseline values. Samples exposed to cyclosporine showed a marked suppression of cytokine expression, demonstrating that the assay worked in a manner consistent with previous studies (Fellman et al., 2011; Riggs et al., 2013).

## 4 DISCUSSION

Pentoxifylline is used to treat several conditions in veterinary medicine. The exact mechanism of action of PTX effects on the immune system has not been established in dogs. An RT‐qPCR based assay to evaluate T‐cell expression of cytokines has been validated and is now routinely used in dogs receiving cyclosporine therapy (Riggs et al., 2013). Because PTX has been shown to alter expression of IL‐2 and IFN‐γ in human studies, and because this potential effect has not yet been investigated in dogs, we chose to use the canine validated RT‐qPCR based assay to assess PTX's effects on these specific cytokines.

A range of PTX concentrations were chosen for this study based on previous pharmacokinetic studies in humans and dogs. Following incubation with differing concentrations of PTX, there were no concentrations that demonstrated a significant difference in ∆Ct values for either cytokine when compared to untreated activated baseline samples. However, when cells were exposed to the highest tested concentration of PTX, the percentage change from untreated activated baseline samples demonstrated a 50% decrease in median cytokine expression for both evaluated cytokines. However, the PTX concentration that was responsible for this effect (200 μg/ml) was over 20 times higher than the maximum peak blood concentrations obtained in pharmacokinetic studies in dogs at standard drug doses. Obtaining a blood concentration of this magnitude in a clinical patient would be highly unlikely at the currently recommended dosages of PTX. Blood samples from one individual dog were, however, noted to consistently exhibit greater than 50% suppression of gene expression of both IL‐2 and IFN‐γ when evaluating percent suppression (Figure 2) at all PTX concentrations, even the lowest concentration assessed, suggesting the possibility of individual dog variation in cytokine expression following exposure to PTX. While the exact mechanisms of action of PTX on the canine immune system are not fully understood, and since a decrease in median IL‐2 and IFN‐γ expression in samples from most dogs only occurred at extremely high and clinically unfeasible PTX concentrations, other potential effects of PTX on the immune system of dogs at the current clinical dosage recommendations should be explored.

Therapeutic drug monitoring is not currently recommended in dogs receiving oral PTX therapy, as target blood concentrations have not been established. Pentoxifylline is typically administered orally within the published recommended dosage range, and the patient is then empirically monitored for a clinical response. Limited PTX pharmacokinetic studies in dogs exist. In one study, the pharmacokinetics of PTX and its metabolites were investigated after oral and intravenous administration in normal dogs (Marsella et al., 2000). Results from this study showed an oral bioavailability for the currently used extended‐release formulation ranging between 15%–32%, which was not affected by the presence of food. Because of a short elimination half‐life, the authors suggested an oral PTX dose of 15 mg/kg every 8 hr in dogs to achieve plasma concentrations similar to those in humans receiving therapeutic dosages. This study documented peak blood concentrations in dogs of 14.3 ± 0.58 µg/ml after intravenous administration. The maximum peak blood concentration when PTX was administered orally was 1.47 ± 0.53 µg/ml, and blood concentrations did not increase with subsequent continued oral dosing. In a second pharmacokinetic study using normal dogs, a Cmax of 10.7 ± 2.7 µg/ml was achieved after intravenous administration of PTX (8 mg/kg as a single injection), and a Cmax of 1.7 ± 0.9 µg/ml was achieved after oral administration of PTX (30 mg/kg as a single oral dose). The authors of this second study considered that PTX concentrations above 1 µg/ml were potentially therapeutic, based on target therapeutic blood values in humans (Rees et al., 2003). All of the PTX concentrations attained in dogs in the two previously discussed studies were markedly lower than the highest PTX concentration used in our study (200 µg/ml), which was the only in vitro concentration to have notable effects on cytokine expression in samples from most dogs. Interestingly, in a human in vitro study, PTX concentrations above 100 µg/ml were associated with complete inhibition of IL‐2 and IFN‐γ (Böhle et al., 2000). These lymphocytes were stimulated with phytohemagglutinin and bacille Calmette‐Guérin, so it is possible that the effects of PTX may depend on the stimulus used.

The decreases in IL‐2 and IFN‐γ expression observed in our study at high PTX concentrations may have the potential to be clinically important, even if those concentrations are higher than those likely to be achieved at standard dose rates, particularly if individual dogs are susceptible to decreased cytokine expression at lower drug concentrations. Anecdotally, veterinarians have administered PTX concurrently with cyclosporine for the treatment of immune‐mediated conditions. Therapeutic drug monitoring for adjusting cyclosporine dosing can include pharmacodynamic assessment, in which activated whole blood IL‐2 expression is measured. If PTX can influence IL‐2 expression at high concentrations, it may have the potential to affect pharmacodynamic assays at lower drug concentrations when used concurrently with cyclosporine, and thereby potentially affect interpretation of the pharmacodynamic assay and influence dosing recommendations. Additional studies are warranted to determine if the concurrent administration of cyclosporine and PTX alters the level of cytokine suppression when utilizing pharmacodynamic monitoring, compared to the administration of cyclosporine alone.

Our study has several limitations. Firstly, our study was conducted as an in vitro study, and it is possible that similar pharmacodynamic testing in dogs receiving oral PTX could have different results. Secondly, our study was conducted in healthy dogs of a single breed, and it is possible that testing in dogs of different breeds and in patients with disease states could produce different results. In vivo studies in healthy dogs as well as testing in dogs of varying breeds and disease states receiving PTX may be warranted.

## CONFLICT OF INTEREST

Drs. Thomason, Mackin, and Archer are affiliated with the Mississippi State University Pharmacodynamic Laboratory, which provides the assay evaluated in this study as a commercial service to veterinarians.

## ETHICAL STATEMENT

The authors confirm that the ethical policies of the journal, as noted on the journal's author guidelines page, have been adhered to and the appropriate ethical review committee approval. The US National Research Council's guidelines for the Care and Use of Laboratory Animals were followed.
